# Sympathetic Surgery.—II

**Published:** 1890-09-27

**Authors:** 


### SYMPATHETIC SURGERY—II.

From the theme of sympathetic growth Sir Kenelm digresses, for no apparent reason, into the endless subject of those stains and swellings in the flesh of children commonly called "mothers' marks." The instances he gives are for the most part such as could be improved on by any *sage-femme* in the land; but around one the charm of a historic mystery dwells. Why was it that King James VI. of Scotland and I. of England had such an aversion from a naked sword—"cauld iron," as he called it in his vernacular? Was it cowardice? We moderns are apt to think so, but this King Jamie's courtiers strenuously denied. They recalled how his mother had seen Rizzio killed before her eyes, and held that the shock of that murder had developed a horror of steel in her unborn child. Nevertheless, it was an unkingly weakness, and one which might have had untoward results to the learned Sir Kenelm himself. For he says: "I remember when he dubbed me Knight, in the ceremony of putting a naked sword upon my shoulder, he could not endure to look upon it, but turned his face another way, insomuch that in lieu of touching my shoulder he had almost thrust the point into my eyes, had not the Duke of Buckingham guided his hand aright."

"But this digression hath already been too long," says Sir Kenelm, after a long discussion of these physiological eccentricities, and therewith he proceeds to digress again. And this fact incites us to indulge in an apothegm: "Beware of the man who apologises for his tediousness, for he seeks to gain from the applause with which your courtesy will greet him an excuse for being tedious again." Do we not all know him? Sometimes he is the parliamentary orator who says, "But I fear I may weary you," and responds to the shuffling of feet that says so plainly, "You do; you do," by wearying his audience for another half an-hour. Sometimes it is the preacher who says, "But my brethren, lest I should detain you too long, let me say in conclusion"—and as sleepy men and languid women rouse themselves at the thought, "He is nearly done!" plunges into the mysterious meaning of the little horn or the feet of clay. Once to our knowledge it was an opera singer, who, having finished a long and dreary *scena*, greeted the mild plaudits that were yielded, more as a tribute to her sex than to her performance, by going through the whole thing over again. Who can avenge us on these criminals? The modest journalist can when he is so inclined. It is a fine thing to his fellow-sufferer to see the reporter lay his watch upon the table and hear him say in a low stern whisper, "If that man goes on for three minutes longer I won't mention him in my report."

For ourselves, we incline to prefer Sir Kenelm's digressions to what he calls the "the great channel and thread of his discourse." But then we are cynics of the nineteenth century, to whom the idea of the sympathetic powder is an exploded fallacy, and who are interested in Sir Kenelm's notions only in so far as they throw light on the science and superstitions of his age. How the solemn assembly of Montpellier, who thought they were on the track of a great discovery, took these meanderings we do not know, but we may reasonably surmise that they regarded them with some impatience. "But what about the powder?" one or two may have murmured as the speaker plunged into the "History of the Tarantula of the Kingdome of Naples." This tarantula is a venomous spider, the effect of whose bite is to make those who have suffered from it mad to dance. So they set about jigging, and keep on with their gyrations till they have danced the poison out of their bodies, and then fall to the earth—exhausted, but safe. It is from this legend of the spider that we get the tarantella, the Neapolitan national dance, and pianoforte pieces innumerable.

"But it is high time," says Sir Kenelm at this point, "that I come to my seventh and last principle." This principle is, "that the source of these spirits, or the body which attracts them to itself, draws likewise after them that which accompanies them, as also that which sticks, and is glued and united with them." How is that proved? By the following domestic experiment. The English housewives of that day, if when heating milk in a saucepan some boiled over and fell on the fire, were wont to throw some salt upon the cinders where the milk had fallen. They believed that if they failed to do this the cow who had given the milk would soon fall ill. And this notion the learned Sir Kenelm accepts as proof of his theory. He admits that some persons—perhaps he had Sir Thomas Brown in his mind's eye—would fancy that "some superstition or folly might lie therein"; but he himself can explain it all by one of his fine-drawn theories. The milk will naturally fly back to the cow who gave it; it will carry with it the fiery particles that burnt it, and thereby set up inflammation. But the salt remedies all this, "because it is of a nature clean, contrary to the fire, the one being hot and volatil, the other cold and fixed, insomuch that where they use to meet, the salt, as it were, knocks down the fire by precipitating and destroying its action." Is not this an eminently satisfactory explanation? And, after this, who shall say that it is impossible to extract sunbeams from cucumbers?

Sir Kenelm, who seems to have had agricultural tastes, plunges yet more deeply into farming mysteries, and shows that the best way of curing an ulcer in an ox's foot is to expose a sod of turf, saturated with putrescent matter that has exuded from the sore, to the cold influence of the north wind. If these things are possible, who shall say that one cannot cure a wound by sprinkling a healing powder on something else! So now, led up to it by these minor cases, we are in a position to profit by the narration of one of the triumphs of sympathetic surgery.

A gentleman of Sir Kenelm Digby's acquaintance, Master Howell by name, was injured in the hand while trying to part the swords of two friends whom some momentary quarrel had driven to fight. The common lot of mediators fell upon him, but he succeeded in calming the combatants, who, shocked at seeing his blood flowing, forgot their quarrel in the attempt to stanch his wound. The bandage most easily attainable was Mr. Howell's garter, with which accordingly the hand was bound up till a surgeon could see to it. Then plaisters were applied, but Mr. Howell got worse, till the pain of his inflamed hand was unendurable. At this juncture he met Sir Kenelm Digby, and told him his case. "I asked him then," says the narrator, "for anything that had the bloud upon it; so he presently sent for his garter, wherewith his hand was first bound, and having called for a bason of water, as if I would wash my hands, I took an handful of powder of vitriol, which I had in my study, and presently dissolved it. As soon as the bloudy garter was brought me I put it within the bason, observing in the interim what Mr. Howell did, who stood talking with a gentleman in a corner of my chamber, not regarding at all what I was doing; but he started suddenly as if he had found some strange alteration in himself. I asked him what he ailed. 'I know not what ailes me, but I find that I feel no more pain; methinks that a pleasing kind of freshnesse, as it were a cold napkin did spread over my hand, which hath taken away the inflammation that tormented me before.' I replied, 'Since that you feel already so good an effect of my medicament, I advise you to cast away all your plaisters, only keep the wound clean and of a moderate temper betwixt heat and cold.'" This was done, and in a few days the hand was well. This was mysterious enough; but while admitting that we cannot explain the sudden relief that Mr. Howell felt when his garter was immersed in water, we are tempted to think that the rest of the cure is explained by the words, "only keep the wound clean." But such an explanation would be far too simple for a subtle-witted philosopher like Sir Kenelm Digby. Rather let all the atoms of the universe meet and join, combine in new alliances, and then fly back to their old homes, than that mere cleanliness should have the credit of performing a cure—cleanliness and the *vis medicatrix naturæ*. Let us mix our vitriol (at eighteenpence a pound) with gum tragacanth, and smear swords and bandages with it. If no cure is wrought, account for the failure by saying the weapon had been heated or the linen washed; but if all go well, praise the powder of sympathy! We are reminded of a doggerel rhyme about homœopathic soup which ends—

"If the patient die say 'twas nature did it,
If he chance to live, give the soup the credit."

We confess to remaining unconvinced by the case of Mr. Howell—the only one Sir Kenelm quotes in his hundred and fifty-two pages. And yet we cannot help seeing throughout his discourse the action of an earnest, keen, and energetic mind. He took a wrong turning when burrowing into the mysteries of nature, and it is his fate to dwell in the memories of men by the thing in which he was wrong rather than by that in which he was right. We have chronicled his errors, but it is not such blundering as his that delays the progress of knowledge. When the earnest student, the conscientious worker falls, "his face is still towards the light." He himself does not always correct the fault, but others coming after see where he went astray. His mistakes are undone in the clearer light of science more complete than he has known, but every inch he won for truth is to the end of time the inalienable heritage of mankind. In these pages we are often proud to chronicle the successes of surgery; let us for once dedicate a word to the failures whence so often that success has sprung. And therefore let us be courteous and respectful in our farewell to that learned and honourable gentleman, Sir Kenelm Digby, and his elaborate discourse on the Powder of Sympathy.

